# Yeast two-hybrid analysis of a human trabecular meshwork cDNA library identified EFEMP2 as a novel PITX2 interacting protein

**Published:** 2012-08-07

**Authors:** Moulinath Acharya, Michael W. Sharp, Farideh Mirzayans, Tim Footz, LiJia Huang, Chanchal Birdi, Michael A. Walter

**Affiliations:** 1 Department of Medical Genetics, University of Alberta, Edmonton, AB, Canada; 2 Department of Ophthalmology, University of Alberta, Edmonton, AB, Canada

## Abstract

**Purpose:**

Mutations in the homeobox transcription factor paired-like homeodomain transcription factor 2 (PITX2) cause Axenfeld–Reiger syndrome (ARS), which is associated with anterior segment dysgenesis (ASD) and glaucoma. To understand ARS pathogenesis, it is essential to know the normal functions of PITX2 and the proteins with which PITX2 interacts in the eye. Therefore, we used a unique cDNA library that we created from human trabecular meshwork (TM) primary cells to discover PITX2-interacting proteins (PIPs).

**Methods:**

A human TM cDNA library was created from primary cells in the ProQuest Two-Hybrid prey vector: pEXP-AD502. Human PITX2A and PITX2C isoforms were used independently as “bait” to identify novel PIPs. A total of 1.25×10^6^ clones were screened by yeast two-hybrid (Y2H) analyses. PIPs obtained from each Y2H experiment were confirmed by yeast retransformation and mammalian co-immunoprecipitation assays.

**Results:**

EGF-containing fibulin-like extracellular matrix protein 2 (EFEMP2) was identified by both PITX2A and PITX2C isoforms as a novel PIP from Y2H analyses. EFEMP2 is 443 amino acids long with six epidermal growth factor (EGF)-like modules and one fibulin-like module. The PITX2-interaction domain in EFEMP2 lies between the second EGF-like module and the COOH-terminal fibulin-like module. Co-immunoprecipitation assays in COS-7 cells confirmed the interaction between PITX2 and EFEMP2.

**Conclusions:**

We discovered EFEMP2 as a novel PITX2-interacting protein. Further, our cDNA library made from human TM primary cells is a unique and effective resource to identify novel interacting proteins for glaucoma and ASD candidates. This resource could be used both for discovery and validation of interactomes identified from in silico analysis.

## Introduction

The paired-like homeodomain transcription factor 2 (PITX2, also known as pituitary homeobox transcription factor 2) is a member of the paired-bicoid class of homeodomain (HD) proteins. Pituitary homeobox transcription factors are involved in a variety of developmental processes including formation of pituitary gland, hind limb and anterior segment of the eye, and brain morphogenesis [1-3]. Expression of PITX2 is found during ocular development [4,5]. Also, a recent report suggests that the expression of PITX2 in adult murine eye regulates the expression of contractile proteins required for proper functioning of extraocular muscle [6].

Mutations in *PITX2* cause Axenfeld-Rieger syndrome (ARS), a group of autosomal dominant clinical disorders affecting anterior eye structures [7]. Classic ocular features of ARS include iridocorneal synechiae, iris hypoplasia, corectopia, polycoria, and/or prominently displaced Schwalbe’s line [8,9]. Approximately 50% of ARS patients develop glaucoma with a great variability in age-of-onset, but usually in the teens [10]. Mild craniofacial dysmorphism, dental defects and/or excessive peri-umbilical skin are considered systemic manifestations often associated with ARS [11]. Congenital cardiac defects and/or hearing loss have been rarely observed in cases of ARS [12]. Generation of mouse models lacking either one (*pitx2^+/−^*) or both copies of *pitx2* (*2pitx2^−/−^*) caused a pleiotropic phenotype that overlaps with the Pitx2 expression pattern and provided a good model for human ARS [13].

Various disease-causing mutations in *PITX2* have been analyzed for multiple cellular and biologic functions including protein stability, DNA binding properties, transcription activation ability and sub-cellular localization [14-16]. Forkhead box c1 (*FOXC1*) is a forkhead box transcription factor that has been associated with ARS when mutated [17,18]. Previous work from our laboratory discovered a physical interaction between PITX2 and FOXC1 where PITX2 acts as a negative regulator of FOXC1 function, indicating that these two transcription factors function in a common pathway [19]. Recently, we identified another novel PITX2-interacting protein, PRKC apoptosis WT1 regulator (PAWR), which inhibits PITX2 transactivation [20]. Taken together these reports indicate that PITX2 is a part of a large network of proteins involved in gene regulation, critically involved in ocular development and disease.

In this report we further validated the utility of our human trabecular meshwork (TM) cell cDNA library by performing two independent yeast two-hybrid screens using two different PITX2 isoforms, PITX2A and PITX2C. Both of these screens revealed EGF-containing fibulin-like extracellular matrix protein 2 (EFEMP2) as a novel PITX2-interacting protein, which we further confirmed by retransformation assay in yeast and co-immunoprecipitation in an immortalized human ocular cell line.

## Methods

### Plasmid constructs

A TM cDNA yeast two hybrid library, created from mRNA extracted from human TM primary cell culture was used independently to identify proteins that interact with the two most abundant PITX2 isoforms found in the eye namely, PITX2A and PITX2C. The making of TM cDNA library inserts and PITX2A and PITX2C “bait” vectors were described previously [20,21]. Briefly, the pCI-PITX2C construct was used as a template for PCR of the open reading frame of PITX2C using PITX2C attB1 (5'-GGG GAC AAG TTT GTA CAA AAA AGC AGG CTT CAT GAA CTG CAT GAA AGG CC-3') and PITX2C attB2 (5'-GGG GAC CAC TTT GTA CAA GAA AGC TGG GTA CAC GGG CCG GTC CAC TG-3'). The PCR product containing attb1 and attB2 sites was subsequently cloned into pDEST32 in-frame using Gateway technology (Invitrogen, Burlington, ON). The V5-EFEMP2_ΔN36_ construct was created by cloning the longest cDNA insert from the Yeast-2-Hybrid (Y2H) screen into the vector pcDNA3.1/V5-DEST (Invitrogen). For full-length EFEMP2 open reading frame (ORF), the *EFEMP2* cDNA clone was obtained from Origene, Rockville, MD, and NheI (5′-GCTAGC-3′) and KpnI (3′-CCATGG-5′) restriction sites were introduced along with the V5 epitope followed by a stop codon by polymerase chain reaction (PCR). This PCR product was first cloned into a pcDNA3.1 plasmid and subsequently into pFLAG-CMV5a plasmid (Sigma-Aldrich, Oakville, ON). Hemagglutinin (HA)-tagged full-length PITX2A and deletion constructs in pCI vector [19] and Xpress-tagged PITX2C in pcDNA4 vector were described previously [22]. Briefly, the WT NH_2_-terminal HA-tagged PITX2A protein isoform was expressed from a cDNA carried in the pCI plasmid (Promega, Madison, WI) and was constructed by subcloning the EcoRI/XbaI fragment from the pcDNA4:PITX2 vector (13) downstream from the HA-epitope sequence (5'-ATG GCT TCT AGC TAT CCT TAT GAC GTG CCT GAC TAT GCC AGC CTG GGA GGA CCT TCT-3') between the NheI/XbaI sites in pCI.

### Yeast two-hybrid analyses

A human TM cDNA library fused to the GAL4AD of pEXP-AD502 (Invitrogen) was screened for proteins that interact with both human PITX2A and PITX2C, using the ProQuest Two-Hybrid System (Invitrogen). The detailed method of yeast two-hybrid screening has been described previously [21]. Briefly, the pEXP-AD502 library plasmid and the pDEST32-PITX2C bait plasmid were co-transformed into MaV203 yeast cells. Transformed yeast cells were plated on medium lacking histidine or uracil or medium containing 5-fluoroorotic acid (5FOA). The transformed yeast cells were also plated on YPAD plates to further conduct β-galactosidase assays. A total of 1×10e6 library clones were screened for growth on selective media and assayed for β-galactosidase activity. pEXP-AD502 cDNA plasmids were recovered by bacterial transformation of DNA isolated from positive yeast colonies. The candidate pEXP-AD502 cDNA plasmids were retransformed into yeast cells with the empty pDEST32 vector or pDEST-PITX2C or pDEST32 plasmid encoding irrelevant bait to exclude false-positives. Inserts of true-positive pEXP-AD502 cDNA clones were characterized by sequence analysis.

### Mammalian cell culture and transfection

HeLa (ATCC, Manassas, VA), COS-7 (ATCC), human trabecular meshwork (TM-1) [23], human corneal endothelium (HCEC; a gift from Dr. Jürgen Bednarz, University of Hamburg), and non-pigmented ciliary epithelium (NPCE; a gift from Dr. Miguel Coca-Prados, Yale School of Medicine) cells were maintained in High or Low Glucose Dulbecco’s modified Eagle’s medium (Invitrogen) supplemented with 10% fetal bovine serum and 1% antibiotic and/or antimycotic agents in a 37 °C, humidified incubator under an atmosphere containing a constant 5% CO_2_. Transfections were performed using FuGene 6 (Roche, Mississauga, ON) or Trans IT-LT1 (Mirus Bio, Madison, WI) according to the manufacturers’ protocols, while 4 μg, 2 μg, or 0.8 μg of total DNA was used for transfections in 100 mm, 60 mm, or 6-well dishes, respectively. Transfected cells were subjected to co-immunoprecipitation assay 48 h after transfection.

### Reverse transcription-polymerase chain reaction (RT–PCR)

Total RNA was extracted from cell lines (TRIzol® Reagent; Invitrogen). A standard 20 μl reverse transcription reaction contained the following components: 1 μg DNaseI-treated total RNA, 500 ng oligo d(T), 0.5 mM (each) dNTPs, 1× First Strand Buffer, 0.01 M DTT, 40 U RNase inhibitor (RNaseOUT; Invitrogen), and 200 U reverse transcriptase (M-MLV; Invitrogen). Replicate reactions without reverse transcriptase were used as negative controls. 0.4 μl of each reaction was used as template for standard 20 μl PCR reactions using the following primer sets: *PITX2* forward 5′-AGG CCA CTT TCC AGA GGA AC-3′; *PITX2* reverse 5′-CGC TCC CTC TTT CTC CAT TT-3′; *EFEMP2* forward 5′-GTG CCT ACA ATG CCT TTC AGA-3′; *EFEMP2* reverse 5′-CAT GAG GGA ATT CAT GGT GAC-3′; hypoxanthine phosphoribosyltransferase 1 (*HPRT1*) forward 5′-GCC AGA CTT TGT TGG ATT TGA-3′; *HPRT1* reverse 5′-GGC TTT GTA TTT TGC TTT TCC AG-3′. Thermal cycling was performed with the following conditions: 95 °C, 3 min; 30 cycles of 95 °C - 30 s, 60 °C - 30 s, 72 °C - 30s. Products were separated in a 2% agarose gel stained with RedSafe™ Nucleic Acid Staining Solution (Frogga Bio, Toronto, ON) and imaged on the Kodak Imagestation 4000MM (Mandel Scientific Company Inc., Guelph, ON).

### Co-immunoprecipitation

Seventy five μl of each cell lysate was diluted with 425 μl of immunoprecipitation (IP) buffer. The detailed method of co-IP has been described previously [19]. Briefly, transfected cells were harvested 3 days after transfection to allow optimal expression of recombinant proteins. Plates were washed in PBS at 25 °C prior to harvest. All subsequent steps were performed at 48 °C in the presence of the mammalian protease inhibitor cocktail (5 ml/ml; Sigma Aldrich). Briefly, cells were scraped from 10 cm dishes in 1 ml of PBS with sterile cell lifters (Corning) and pelleted via microcentrifugation at 3,000× g for 10 min. The pellet supernatant was removed and a whole-cell lysate obtained by suspending the pellet in 200 ml RIPA buffer (1% PBS, 1% [v/v] IGEPAL CA-630, 0.5% [w/v] sodium deoxycholate, 0.1% [w/v] SDS). Samples were vortexed briefly and incubated on ice for 45 min to allow maximal nuclear lysis. Lysates were cleared of cell debris by microcentrifugation at 18,000× g for 10 min and total protein concentration was calculated (Bio-Rad). Lysate concentrations were equalized in a fixed volume of RIPA prior to immunoblot analysis of immunoprecipitation inputs. The antibody-protein complex was eluted in standard 2× SDS loading buffer followed by sodium dodecyl sulfate-polyacrylamide gel electrophoresis (SDS–PAGE) and immunoblot analyses using anti-Xpress and anti-V5 antibodies (Invitrogen).

## Results

### Identification of EFEMP2 as a PITX2-interacting protein using Y2H screening

**Figure 1:**
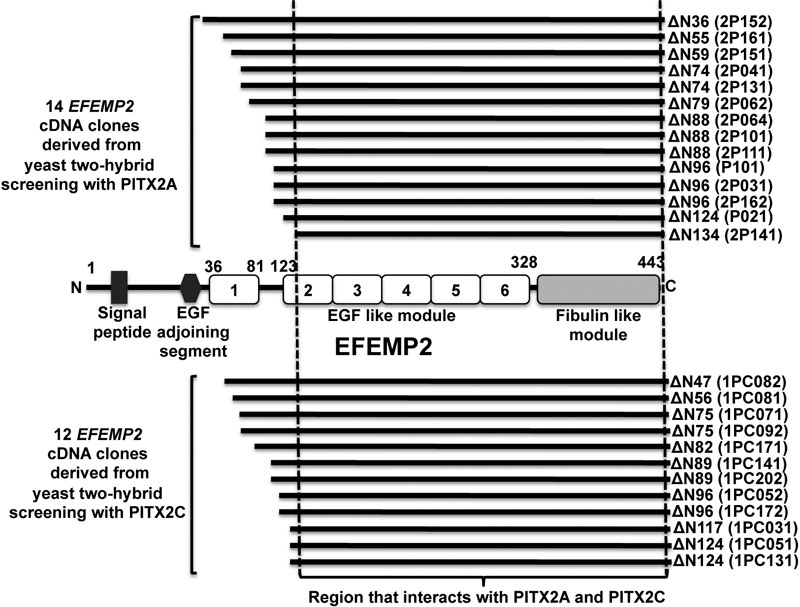
Summary of the yeast two-hybrid analysis using PITX2A and PITX2C as “bait.” EFEMP2 was identified as a novel PITX2-interacting protein by independent yeast two-hybrid (Y2H) analysis with two commonly expressed PITX2 isoforms. Y2H screening with PITX2A resulted in 14 *EFEMP2* cDNAs from approximately 250,000 yeast clones while 12 *EFEMP2* cDNAs were derived from screening approximately one million yeast clones with PITX2C. Combining all positives, the largest *EFEMP2* clone lacks the NH_2_-terminal 36 amino acid residues while the smallest *EFEMP2* clone lacks the NH_2_-terminal 134 amino acid residues. A schematic representation of EFEMP2 protein therefore shows that the PITX2 interacting domain in EFEMP2 lies between the second EGF-like module and the COOH-terminal fibulin-like module (135 to 443 amino acid residues).

**Figure 2:**
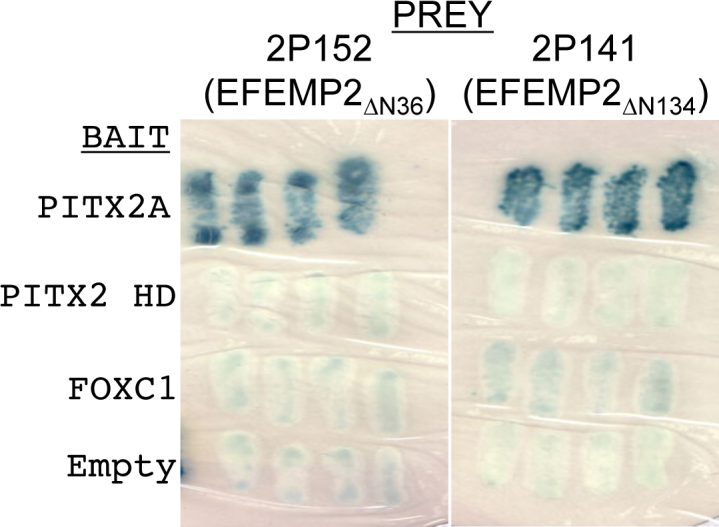
Retransformation assay and test against other bait constructs. The 2P152 and 2P141 prey constructs were re-co-transformed into yeast with the original PITX2 bait construct and the interaction phenotypes were re-assessed. The results of the X-gal assay are shown here. Blue colonies indicate positive interaction while white colonies indicate no interaction. The 2P152 and 2P141 prey constructs were also tested for interactions with the PITX2 homeodomain bait construct, and a full length FOXC1 bait construct, both yielding negative results. The 2P152 and 2P141 prey constructs were also co-transformed with an empty bait construct to test for prey self-activation.

**Figure 3:**
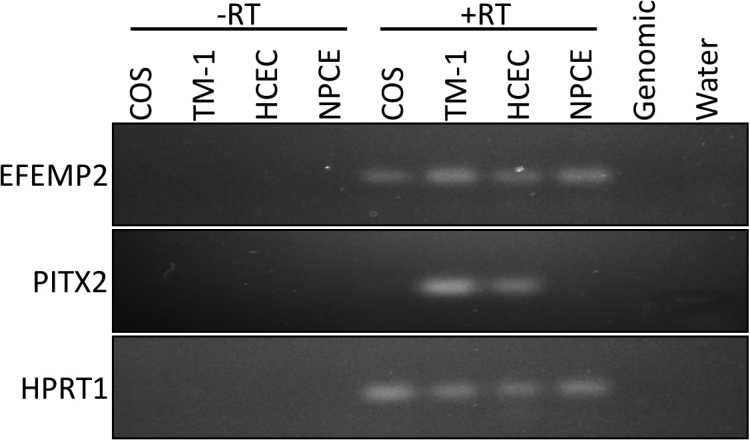
Detection of EFEMP2 and PITX2 expression in ocular cell lines by RT–PCR. Intron-flanking primers were used to amplify mRNA transcripts of *EFEMP2* (161 bp) and *PITX2* (133 bp) from various transformed cell lines, using the housekeeping gene *HPRT1* (130 bp) as a control. Reverse-transcribed (RT) samples either contained (+) or lacked (-) enzyme, and control templates of genomic DNA and water were included, to verify that amplified products were a result of specific primer binding.

A total of 1.25×10^6^ clones (human TM cDNA Y2H library) were screened using PITX2A and PITX2C as “bait.” A total of 32 independent clones (16 for each PITX2A and PITX2C) were obtained that fulfilled the criteria of interaction between gene products. Isolation of plasmids followed by partial sequencing using vector specific primer was done for all 32 clones. Out of 16 clones that interacted with PITX2A, 14 were found to match with cDNAs for EGF-containing fibulin-like extracellular matrix protein-2 (*EFEMP2*, OMIM 604633; Figure 1), one was false positive and another was determined to be an out of frame cDNA for the mannose-6-phosphate receptor binding protein. For *PITX2C*, out of 16 clones, 12 were *EFEMP2* (Figure 1), two clones were false-positive while the remaining two were PRKC apoptosis WT-1 regulator (*PAWR*) cDNA sequences [20] For both *PITX2A* and *PITX2C*, the longest interacting *EFEMP2* clone lacks the first 36 amino acids while the shortest lacks the first 134 amino acids from the NH_2_-terminus (Figure 1). The specificity of the interaction between EFEMP2 (protein encoded by *EFEMP2*) with both PITX2A and PITX2C was confirmed by retransformation of the positive cDNA clone into yeast cells (Figure 2). To investigate the ocular expression pattern of *EFEMP2*, we performed RT–PCR on available human cell line RNA and detected its transcript in trabecular meshwork, corneal endothelium and non-pigmented ciliary epithelium (Figure 3), overlapping with detectable expression of PITX2 in the TM-1 and HCEC lines. A computer search for mouse Efemp2 in annotated microarray results at revealed expression in additional ocular tissues including retina, iris, eyecup, and lens (data not shown).

### PITX2 and EFEMP2 interact with each other in mammalian cells

**Figure 4:**
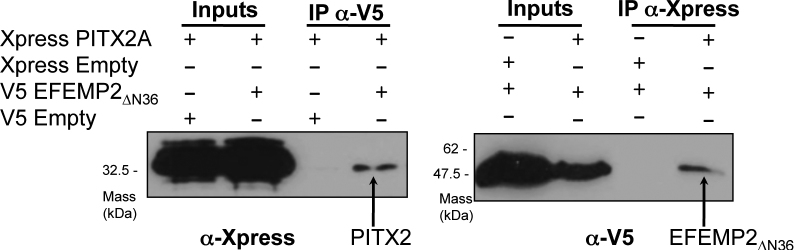
Confirmation of interaction between PITX2 and EFEMP2. Left Panel: The Xpress-PITX2A protein was immunoprecipitated with anti-Xpress antibodies and V5-EFEMP2_ΔN36_ was detected with anti-V5 antibody. V5-EFEMP2_ΔN36_ was not immunoprecipitated in the absence of the PITX2 (XpressEmpty control). Right Panel: In a reciprocal experiment, V5-EFEMP2_ΔN36_ was immunoprecipitated with anti-V5 antibodies and Xpress-PITX2A was detected with anti-Xpress antibody. Similarly, Xpress-PITX2 was not immunoprecipitated in the absence of the EFEMP2(V5Empty control). Twenty percent of the amount of cell lysate used for immunoprecipitation was loaded on the SDS–PAGE for the “input” lanes.

The interaction between PITX2 and EFEMP2 was further confirmed by co-immunoprecipitation experiments where COS-7 cells were co-transfected with Xpress tagged PITX2 (pcDNA vector) and either empty pcDNA3.1 vector or V5-EFEMP2_ΔN36_. Immunoprecipitation using the anti-Xpress antibody (Invitrogen) to Xpress-tagged PITX2 followed by immunoblotting using the anti-V5 antibody (Invitrogen) resulted in co-purification of V5-EFEMP2_ΔN36_ in co-transfected cells (Figure 4, left panel). In a reciprocal experiment using V5-tagged EFEMP2_ΔN36_ co-transfected with either empty pcDNA4 vector or Xpress-tagged PITX2, Xpress-tagged PITX2 was co-purified in co-transfected cells (Figure 4, right panel).

### PITX2 interacts with EFEMP2 through the PITX2 NH_2_-terminal region adjacent to HD

**Figure 5:**
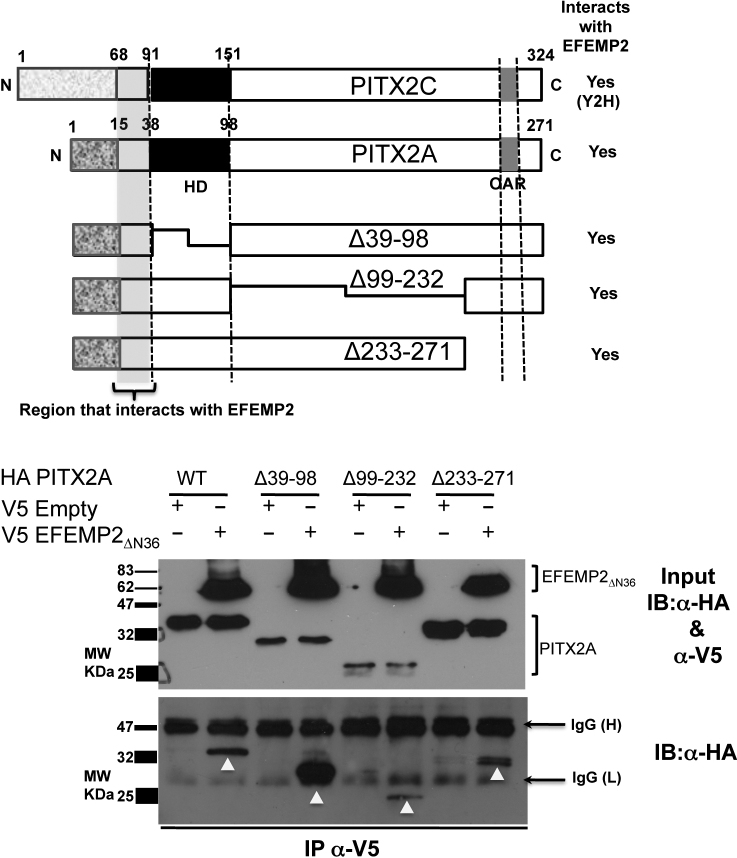
Identification of the region in PITX2 that interacts with EFEMP2. Upper panel: Schematic representation of full-length PITX2C, PITX2A and the PITX2 deletion constructs used in this study. Lower Panel: The HA-PITX2A wild type (WT) and deletion constructs (Δ39–98, Δ99–232 and Δ233–271) were tested for interaction with EFEMP2_ΔN36_ using co-immunoprecipitation experiments. All PITX2 constructs (WT, Δ39–98, Δ99–232 and Δ233–271) bound to V5- EFEMP2_ΔN36_ were immunoprecipitated with anti-V5 antibody and detected subsequently by immunoblotting using anti-HA antibody. This experiment confirms that the all PITX2 constructs used in this experiment interact with EFEMP2_ΔN36_ suggesting that the NH_2_-terminal region before the homeodomain in PITX2, commonly shared by all these PITX2 constructs interacts with EFEMP2_N36_. Inputs represent 20% of the cell lysates used for immunoprecipitation experiments.

Co-immunoprecipitation assays were performed to identify the specific region of PITX2 that interacts with EFEMP2. Full-length and deletion constructs of PITX2 (pCI-HA) [19] were transfected into COS-7 cells along with either empty pcDNA3.1 vector or pcDNA3.1-V5-EFEMP2_ΔN36_ (Figure 5). All PITX2 deletion constructs used in this study (Δ39–98, Δ99–232 and Δ233–271) were found to interact with EFEMP2 (Figure 5). The only region in common between these PITX2 deletion constructs is the NH_2_-terminal region adjacent to the HD, indicating that this PITX2 region interacts with EFEMP2. Consistent with this inference, the NH_2_-terminal 23 amino acid residues before the HD is commonly shared between the PITX2A and PITX2C isoforms, which both interact with EFEMP2 in the Y2H assays. We have therefore deduced that this small region is the EFEMP2-interacting domain of PITX2 (Figure 5).

## Discussion

PITX2 was identified as the first candidate gene to cause ARS [5]. However, knowledge of exactly how *PITX2* mutations cause ARS is still lacking. Identification and characterization of new PITX2-interacting proteins (PIPs) is therefore necessary to better understand the PITX2 regulatory network in the eye. In this report we identified EFEMP2 (fibulin-4) as a novel PIP using Y2H screening of a human trabecular meshwork cDNA library independently with PITX2A and PITX2C isoforms. *EFEMP2* belongs to the *FIBULIN* family of genes that consists of five known extracellular matrix proteins. These proteins can be further subdivided into two groups. The first group consists of fibulin-1 and fibulin-2. These two proteins have an anaphylatoxin domain on their NH_2_-termini. Fibulin-1 and fibulin-2 are also slightly larger than the proteins in the second group (~90–100 kDa and 200 kDa, respectively) [24]. The second group is composed of fibulin-3, −4, and −5, which are 50–60 kDa in size [25]. Fibulins belong to the epidermal growth factor (EGF) superfamily, which include members like hemicentin (also called fibulin-6) and fibrillin proteins. The fibulins have EGF domains that span 35–45 amino acids. EFEMP2 has six EGF domains and a fibulin-like domain. Among the EGF domains, EGF-1 is atypical while the others have potential calcium-binding domains. PITX2 and EFEMP2 have a large overlap in tissue expression. *EFEMP2* mRNA is expressed in human tissues that include the heart, eye, brain, pituitary gland, lung, and kidney [25-27]. Both *PITX2* and *EFEMP2* were detected by RT–PCR in trabecular meshwork and cornea cell lines (Figure 3), and their expressed sequence tags (ESTs) have been identified in different ocular tissues including the TM, iris, optic nerve, anterior segment, and fetal eyes (UniGene EST data). *Efemp2* is expressed as early as day 7 in developing mouse embryos and *Pitx2* is expressed as early as day 10.5 [25,26,28]. Thus, *PITX2* and *EFEMP2* exhibit an overlapping expression profile, both in fetal and adult mammals.

The interaction between PITX2 and EFEMP2 proteins was verified at a molecular level in reciprocal co-immunoprecipation reactions (Figure 4). Further, combining our Y2H analysis and domain mapping analyses, we discovered that the NH_2_-terminal 23 amino acid residues adjacent to the HD of PITX2 is the interaction domain for EFEMP2 (Figure 5). While other regions in PITX2 including the HD and OAR domain have been shown to interact with several other proteins such as FOXC1 and PAWR [19,20], this is the first demonstration that the PITX2 N-terminal domain is involved in protein–protein interaction.

While mutation of *EFEMP2* does not appear to be a direct cause of general Anterior Segment Dysgenesis (ASD; unpublished data), it remains an interesting candidate gene for glaucoma and other anterior segment diseases. Mutations in *EFEMP2* have been associated with cutis laxa, an autosomal recessive disorder of connective tissue characterized by inelastic tissue in all affected areas of the body [29]. Recently, the related fibulin-5 has been shown to inhibit cell proliferation and migration of choroidal endothelial cells and downregulates mRNA expression of vascular endothelial growth factor (*VEGF*), C-X-C chemokine receptor type 4 (*CXCR4*), and transforming growth factor β1 (*TGFB1*), thus implicating fibulin-5 in choroidal neovascularization and neovascular age-related macular degeneration (AMD) [30]. These findings lead us to speculate that EFEMP2 may also play a pleiotropic role in ocular disorders by affecting signaling in the ECM. *EFEMP2* is located at chromosome 11q13, in close proximity to the nanophthalmos (NNO) 1 locus. NNO is inherited in autosomal dominant and recessive manners [31-33] accompanied with anterior chamber defects and an increased rate of glaucoma [34]. Since the gene for NNO1 has not been identified, it may be useful to screen *EFEMP2* in a panel of NNO patients. Therefore, a properly designed screening effort would be important and worthwhile in terms of investigating the involvement of *EFEMP2* in ocular diseases including ARS and NNO.

In this study we interrogated our unique and useful resource of a human trabecular meshwork cDNA library with bait constructs of different protein isoforms of PITX2 to discover a novel PITX2-interacting protein, EFEMP2, which indicates the precision and quality of this cDNA library. This resource could be very useful and important to identify novel protein–protein interactions in glaucoma and other developmental eye anomalies considering the functional importance of trabecular meshwork in the eye anterior segment.
